# Evaluating a Proprietary Tannin-Blend Product as an Alternative to Monensin and Tylosin Phosphate in Feedlot Cattle Diets

**DOI:** 10.3390/vetsci12050446

**Published:** 2025-05-06

**Authors:** Luana D. Felizari, Luke K. Fuerniss, Jonathan L. Beckett, David S. Secrist, Guy D. Hufstedler, Bradley J. Johnson

**Affiliations:** 1 Department of Animal and Food Sciences, Texas Tech University, Lubbock, TX 79409, USA; luana.felizari@ttu.edu (L.D.F.); fuernissl@gmail.com (L.K.F.); 2 Beckett Consulting Services, Fort Collins, CO 80524, USA; jbeckett@beefconsulting.com; 3 Department of Animal, Dairy, and Veterinary Sciences, Utah State University, Logan, UT 84322, USA; david.secrist@usu.edu; 4 Silvateam USA, New York, NY 10022, USA; dhufstedler@silvateam.com

**Keywords:** beef cattle, tannin, saponins, monensin, tylosin, liver abscess, performance

## Abstract

This study evaluates the effects of a proprietary tannin blend (BX), supplemented with or without sodium monensin (MON), on beef cattle performance, carcass traits, and health. Steers (*n* = 2986; initial shrunk body weight (SBW) 254 ± 9.2 kg) were allocated into 48 pens (61–62 steers/pen; 12 pens/treatment) fed for 230 d. Treatments included: (1) no feed additives (CTL); (2) BX (7.95 g/animal daily); (3) MON + Tylosin phosphate (TYL; 437.52 mg MON/animal daily + 80 mg TYL/animal daily); or (4) MON + BX (437.52 mg MON/animal daily + 7.95 g BX/animal daily). Data were analyzed in R 4.2.1 using a randomized block design with pen as the experimental unit. Dry matter intake was lower (*p* < 0.001) in MON + TYL and MON + BX than in CTL. Steers fed MON + BX had greater carcass-adjusted final SBW (*p* = 0.002), average daily gain (*p* = 0.002), fat thickness (*p* = 0.035), and marbling score (*p* = 0.046) than BX. Feed conversion improved in MON + TYL and MON + BX (*p* < 0.001). CLT and BX had higher (*p* < 0.001) liver abscess prevalence compared to MON + BX and MON + TYL. The addition of BX did not improve the parameters measured. Steers fed MON + BX showed reduced liver abscesses and similar feed efficiency compared to MON + TYL.

## Simple Summary

Natural feed additives, such as tannins and saponins, are being explored as alternatives to traditional ionophores and antibiotics in feedlot cattle diets. This study aimed to evaluate the effects of a proprietary tannin blend, supplemented with or without sodium monensin, on performance, carcass traits, and health of beef cattle finished in a commercial feedlot. The results indicated that feeding a tannin blend alone did not improve overall growth, carcass traits, or health. However, tannin blend with monensin could be a strategy to improve feed efficiency and reduce liver abscesses prevalence.

## 1. Introduction

High-energy diets are commonly used in feedlot cattle operations to maximize growth and carcass yield. However, feeding high-energy diets increases the risk of metabolic disorders, including ruminal acidosis, laminitis, and bloat, particularly when proper management or a gradual transition to the finishing diet is not implemented [1]. Rumen acidosis can compromise the rumen environment, creating conditions that allow bacteria to translocate from the rumen into the bloodstream. Additionally, “leaky gut” syndrome in the small intestine has also been associated with bacterial translocation to the bloodstream [2]. As a result, bacteria such as *Fusobacterium necrophorum*, *Trueperella pyogenes*, and *Salmonella enterica* may reach the liver, increasing the risk of liver abscesses (LA) [3]. Feed additives are commonly used in feedlot operations to mitigate metabolic disorders and promote growth [4]. The feed additives can be classified into ionophores, non-ionophore antibiotics, organic acids, microbials, and natural plant extracts. According to a survey conducted in the United States, ionophores are included in approximately 97.3% of the feedlot cattle diets, as recommended by nutritionists [5]. Among the ionophores, monensin (MON) is the most frequently used due to its ability to improve average daily gain (ADG), reduce dry matter intake (DMI), and enhance feed efficiency [4,6]. In addition, tylosin phosphate is an antibiotic targeting Gram-positive bacteria, including *Fusobacterium necrophorum*, the principal pathogen associated with liver abscesses [7]. Therefore, tylosin phosphate is commonly used to reduce the incidence of liver abscesses in cattle, potentially lowering prevalence from 30% to 8% [8,9].

Despite the extensive use of ionophores and non-ionophore antibiotics, pressure from society and regulatory policies has grown around antimicrobial resistance (AMR). The European Union banned antibiotic growth promoters in 2006 [10], prompting an increased search for natural alternatives in livestock production. In addition, the macrolide family of antimicrobials, such as tylosin phosphate, is classified as medically important for human health and requires veterinary oversight for use in animal feed. While tylosin phosphate itself is not prescribed in human medicine, its use in livestock may contribute to cross-resistance with other macrolide antibiotics, as it can select for bacteria carrying resistance genes that confer cross-resistance to multiple macrolides important for human health [11]. The World Health Organization recommends no use of antimicrobials in food animals for disease prevention or growth promotion due to concerns about AMR [12]. One of the alternatives is to use plant-based feed additives, such as tannins and saponins, which have the potential to improve the performance and health of cattle without contributing to antimicrobial resistance. Tannins, polyphenolic compounds extracted from plants, are frequently used in ruminant diets and can be classified as condensed tannins (CT) and hydrolyzable tannins (HT). Condensed tannins exhibit stronger antimicrobial properties than HT due to their oligomeric, polymeric proanthocyanidins structure and carbon-carbon bonds, making them more resistant to hydrolysis and degradation [13]. A key characteristic of tannins is their ability to bind to dietary proteins in the rumen, forming complexes resistant to microbial degradation. This process increases the availability of amino acids for the animal [14,15] while decreasing the nitrogen urinary excretion [16]. In addition, tannins contribute to methane mitigation by suppressing microbial hydrolysis, thus decreasing hydrogen availability in the rumen [13,17]. Furthermore, the supplementation of tannins is associated with antioxidant properties and improved immune function in animals, as well as reduced biohydrogenation of unsaturated fatty acids in the rumen [18,19,20]. Similarly, saponins, classified as secondary plant metabolites, have been associated with antimicrobial properties, primarily through the disruption of Gram-negative bacterial cell membranes by interacting with membrane sterols [21]. This mechanism can also suppress populations of ruminal protozoa and associated methanogens, contributing to shifts in microbial composition and reduced methane production [22].

Studies have shown that tannins as a feed additive can improve final body weight (BW), ADG, and/or DMI [23,24,25,26,27,28], although other findings suggest divergent results on cattle performance [29,30,31,32,33]. The combined use of MON and tannins has gained attention. A recent study reported that cattle consuming MON with tannin and saponins had a higher final BW and improved ADG and feed conversion than those fed MON alone [32]. Similarly, combining MON and tannins has been associated with better feed efficiency during the feedlot phase [25]. However, limited studies have investigated the long-term use of tannins and saponins or their combination with MON. As a result, feeding tannins and saponins may reduce metabolic disorders and liver abscesses (LA), improving animal health and performance.

It was hypothesized that supplementation with a proprietary tannin blend, either alone or in combination with sodium monensin, would improve growth performance and reduce liver abscess prevalence in feedlot cattle. Additionally, the tannin blend was considered as a potential alternative to the conventional combination of monensin and tylosin phosphate. The objective of this study was to evaluate the effects of a proprietary tannin blend, supplemented with or without sodium monensin, on the performance, carcass traits, and health of beef cattle finished in a commercial feedlot.

## 2. Materials and Methods

### 2.1. Animal Care and Use

The following experiment was a collaborative study conducted at a commercial cattle feeding facility located in Southwest Kansas. The experiment followed the guidelines stated in the Guide for the Care and Use of Agricultural Animals in Agricultural Research and Teaching [34].

### 2.2. Animals and Treatments

A total of 2986 commercial steers, with initial SBW of 254 ± 9.2 kg, were blocked by initial BW and origin, then randomly allocated into 48 pens (12 pens per treatment; approximately 0.5 m linear bunk space; 23 m^2^ of pen space per animal; soil-surfaced floors; *n* = 62 or 63 animals per pen) according to one of four treatment groups: (1) Control (**CTL**), a basal diet with no inclusion of monensin, tylosin phosphate, or a proprietary blend of tannins and saponins; (2) A proprietary blend of tannins and saponins (**BX**; 7.95 g per animal daily; composed of a proprietary blend of condensed tannin extract from Quebracho (*Schinopsis lorentzii*), hydrolysable tannin extract from chestnut (*Castanea sativa*) and carriers from cereals rich in saponins; Silvateam S.p.A, San Michele Mondovì, Italy; [0.08% of diet DM]); (3) Monensin (MON; 437.52 mg per animal daily; Rumensin™90, Elanco, Greenfield, IN) + Tylosin phosphate (TYL; 80 mg per animal daily; Tylan 100, Elanco, Greenfield, IN; **MON + TYL**); or (4) 437.52 mg of MON per animal daily + 7.95 g of BX per animal daily (**MON + BX**). Cattle began entering the commercial feedlot in November 2020, with the final group arriving in January 2021.

### 2.3. Feeding and Management Description

At the beginning of the experiment, the animals were vaccinated against viral pathogens using Pyramid 5 + Preponse^®^ (Bovine herpes virus type 1, bovine viral diarrhea virus type 1 and 2, bovine respiratory syncytial virus, and parainfluenza-3 virus; Boehringer Ingelheim Animal Health USA, Inc., St. Joseph, MO, USA), treated for internal and external parasites using Safe-Guard^®^ Drench (Merck, Madison, NJ, USA) and Dectomax^®^ Injectable (Zoetis, Kalamazoo, MI, USA). Steers were implanted on day 0 (Revalor-IS; 80 mg TBA + 16 mg E_2_; Merck Animal Health), followed by a reimplant on day 133 (Component TE-S; 120 mg TBA + 24 mg E_2_; Elanco, Greenfield, IN, USA).

The study had a duration of 230 days, including a 21-day adaptation period and a 13-day transition period using a two-ration blend. The basal diet during the finishing phase was composed of flaked corn, high-moisture corn, triticale silage, palm oil, distillers’ grains (DDG), wheat straw, alfalfa hay, and supplement, as shown in Table 1. The ration analyses were conducted using wet chemistry by a commercial laboratory (Servitech, Dodge City, KS, USA). During the finishing phase, due to fluctuations in flaked corn prices, animals were initially fed a flaked corn-based diet before gradually transitioning to a flaked wheat diet over a three-week period. Steers remained on the flaked wheat diet for approximately three months before transitioning back to a flaked corn-based diet for the remainder of the study. The feed additives were incorporated using water as a carrier through a micro-machine system. Animals were fed twice daily under slick bunk management and with continuous access to fresh water in frost-free tanks. Steers were transported to the packing plant (Cargill, Dodge City, KS, USA) and harvested by weight group within a block after 230 days on feed. Four groups of steers, one from each treatment and from the same weight group, were shipped together on the same day for harvest.

### 2.4. Feedlot Performance Measures

The animals were individually weighed at the beginning of the experiment (day 0) and again on the day they were reimplanted (day 133). A 4% shrink adjustment was applied to determine the initial and reimplanted SBW. The dry matter intake (DMI) was recorded on a pen level and calculated as an average across the steers within the pen. The average daily gain (ADG) and feed conversion ratio (F:G) were calculated at the end of the study. Carcass-adjusted performance was determined by dividing the hot carcass weight (HCW) by the dressing percentage (64%), and a 4% shrink adjustment was applied.

### 2.5. Health Response Measures

Steers were monitored daily by a trained individual for signs of morbidity, including respiratory disease, bloat, lameness, or other ailments. Animals exhibiting signs of illness or suspected of being morbid were removed from their pens for examination in accordance with the facility management procedure, with their identification duly recorded. When required, therapeutic interventions were administered under the guidance of a licensed veterinarian. For animals that died during the study period, the cause of death was recorded through necropsy.

### 2.6. Carcass Trait Measures

Steers were transported to a commercial packing plant for harvest and data collection. Following exsanguination, the tag of each animal was recorded to match it with an individual treatment. Immediately after evisceration, livers were scored by a trained evaluator using the Elanco Liver Check System (Greenfield, IN, USA) on a scale of 0 (no abscesses), A- (1 to 2 small abscesses), A (2 to 4 small active abscesses), and A+ (1 or more large active abscesses). Kidney, pelvic, and heart fat (KPH) was assessed by a trained evaluator, and hot carcass weight (HCW) was recorded after the KPH removal. Following a 24 h chill, a certified USDA inspector evaluated the quality grade along with the 12th rib fat thickness, longissimus muscle area, and marbling score. Yield grade (YG) was calculated by the USDA YG equation [35].

### 2.7. Fecal Starch

Pen-level fecal samples were randomly selected to represent the pen during sample collection. Fecal samples were collected during the finishing period in the morning (~06:00 h) from the ground after the animals defecated. The samples were combined for each pen and analyzed for total fecal starch by a commercial laboratory (ServiTech Labs, Amarillo, TX, USA).

### 2.8. Statistical Analysis

All performance data was analyzed and reported on a Deaths-In basis. The statistical analysis was conducted by one-way ANOVA using RStudio 4.2.1 (RStudio; Boston, MA, USA). The data were tested for normality using the Shapiro–Wilk test and for homogeneity of variance using Levene’s test before applying the ANOVA. When ANOVA assumptions were met, Tukey’s Honestly Significant Difference (HSD) test was used for post hoc pairwise comparisons (*p* ≤ 0.05). The experimental design followed a randomized complete block design, with a pen as the experimental unit. Models included treatment as a fixed effect, block as a random effect, and strata as a random effect nested within block. Morbidity and mortality data were analyzed as binary traits using logistic regression with a model that included treatment as a fixed effect, block as a random effect, and strata as a random effect nested within block. Pairwise comparisons were performed when significant differences were detected (*p* ≤ 0.05) in ANOVA. For variables that violated ANOVA assumptions, a non-parametric Kruskal–Wallis test was applied. Distributions of categorical variables, such as liver score and quality grade, were analyzed using the Chi-square (χ^2^) test.

## 3. Results

### 3.1. Feedlot Performance

No significant treatment effects were observed for initial SBW (*p* = 0.499; Table 2) and reimplant SBW (*p* = 0.265). However, steers fed MON + BX had significantly heavier carcass-adjusted final weights (*p* = 0.002) and greater ADG (*p* = 0.002) compared to those receiving BX, with other treatments being intermediate. Animals fed CTL had greater DMI compared to steers fed MON + TYL and MON + BX, both for overall DMI (*p* < 0.001) and DMI from day 0 to reimplant (*p* < 0.001). During the period from reimplant to harvest, CTL also had greater DMI compared to MON + BX. Therefore, the F:G was lower for steers fed MON + TYL and MON + BX compared to CTL and BX, both for the entire feeding period (*p* < 0.001) and from day 0 to reimplant (*p* < 0.001). These differences in F:G indicate that steers fed with MON + TYL and MON + BX had better efficiency compared to the other treatments.

### 3.2. Health

No differences were observed among treatments for morbidity (*p* = 0.898; Table 3) and mortality (*p* = 0.452). Overall, respiratory morbidity showed no significant differences between the treatments (*p* = 0.847). Similarly, the frequency of animals pulled for respiratory disorders requiring first, second, or third treatment did not differ between treatments (*p* > 0.05). No significant effects of treatments were observed for bloat (*p* = 0.559). In addition, no significant differences were observed in mortality rates attributed to respiratory (*p* = 0.537) and digestive (*p* = 0.390) disorders.

### 3.3. Carcass Characteristics

Steers fed MON + BX had heavier HCW (*p* = 0.001; Table 4) compared to those receiving BX, with MON + TYL and CTL being intermediate. Animals fed BX alone demonstrated a slightly more favorable yield grade (*p* = 0.004), compared to those fed MON + TYL and MON + BX. Cattle fed MON + BX had greater fat thickness (*p* = 0.035) and marbling score (*p* = 0.046) compared to animals receiving BX. A lesser prevalence of liver abscesses (*p* < 0.001) was observed in steers fed MON + TYL compared to all other treatments, while MON + BX also showed a significant reduction of liver abscess prevalence compared to CTL and BX. Moreover, animals fed MON + TYL had fewer A+ and A scores (*p* < 0.001) across treatments. No significant effects of treatments were observed for ribeye area (*p* = 0.198), KPH (*p* = 0.060), or quality grade distribution (*p* = 0.073).

### 3.4. Fecal Starch

**Figure 1:**
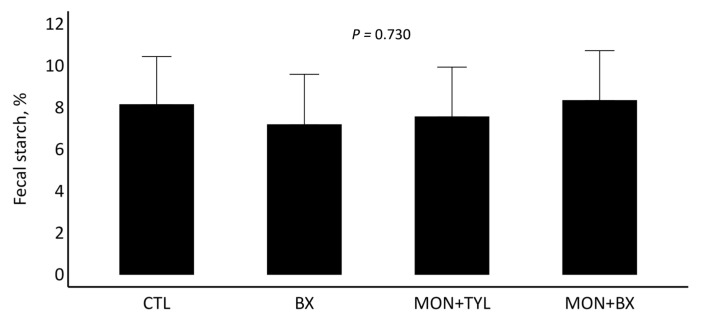
Fecal starch of steers supplemented with a proprietary tannin blend, with or without sodium monensin, or with the combination of sodium monensin and tylosin phosphate during the finishing period.

No significant differences were observed between treatments *(p* = 0.730; Figure 1) for fecal starch percentage during the finishing phase.

## 4. Discussion

As there continues to be significant public concern about the use of antibiotics in cattle production for growth performance, there is an increasing interest in feed additive alternatives that maintain efficiency without relying on the use of in-feed antibiotics. In this study, we evaluated the effects of a proprietary tannin blend, supplemented with or without sodium monensin, on the performance, carcass traits, and health of beef cattle finished in a commercial feedlot.

Tannins are known for their dose-dependent antinutritional properties, including low palatability and reduced nutrient digestion, which can negatively affect cattle performance, generally associated with higher doses. However, at lower-level feed inclusion, tannins can have beneficial effects by protecting dietary proteins from the degradation of the rumen and reducing methane emissions [13,14,15,16,17]. Studies have shown that supplementing diets with tannins at lower levels, below 3% of DM, does not negatively impact DMI in cattle [36,37]. However, higher doses of tannins may decrease DMI and affect ruminal nutrient digestion [14,38,39]. Saponins can also modulate the rumen microbial population, which may decrease metabolic disorders [21].

In our study, DMI in steers fed BX was slightly lower than in the CTL group but not significantly different, indicating that BX did not negatively affect intake or palatability under the conditions of this study. As expected, animals supplemented with MON in combination treatments (MON + TYL and MON + BX) had decreased DMI and improved F:G compared to CTL, aligning with the well-documented effects of monensin on intake reduction and feed efficiency enhancement [4]. These improvements are relevant for producers, as they translate into more efficient nutrient utilization and potentially lower feed costs. In addition, we found that MON + BX supplementation improved carcass-adjusted final BW and ADG compared to BX alone, and BX supplementation did not significantly increase ADG or carcass-adjusted final BW. While other studies observed an increase in ADG feeding tannins [23,24], our results are aligned with previous studies where Nellore bulls fed a blend of tannins and saponins did not significantly affect DMI, ADG, and carcass characteristics [40]. Similarly, no significant differences in DMI were observed across various levels of tannin supplementation (0.0%, 0.5%, 1.0%, and 1.5% of DM) in Nellore and Holstein cattle [41] or when dairy cows were supplemented with tannins at 0.6% of DM [42]. However, a study reported that different tannin levels (0.9% and 1.8% of DM) reduced DMI in dairy cows [43]. Also, aligning with our study, an investigation of a combination of MON and a blend of tannin and saponins showed an improvement in performance, including increases of 2% in final BW and 5.4% in ADG, compared to MON alone [32].

Along with changes in feedlot performance, dietary supplementation also affected carcass characteristics in our study. Specifically, steers fed with MON + BX improved HCW, fat thickness, and marbling score compared to cattle fed BX alone. On the other hand, animals supplemented with BX resulted in better YG compared to MON + TYL and MON + BX. Although studies have not shown the potential improvement in YG feeding tannin [29,33,44], our study diverges and may suggest that BX can influence the carcass characteristics of steers during the feedlot period. Previous studies support the potential benefits of combining a tannin blend with monensin to enhance cattle carcass characteristics. A recent study reported a 3% increase in HCW, a 6.5% improvement in carcass gain, and a 6.09 cm^2^ increase in the gluteus medius area in cattle fed a combination of tannins and MON [25]. However, another study reported that there was no difference in HCW in Nellore bulls fed a blend of tannin and saponins [32]. This suggests that the response to the supplementation may depend on factors, including breed, diet ingredients, or inclusion levels. Although we observed no differences in most of the results of carcass characteristics between BX and CTL treatments, other studies that supplemented BX found increased HCW and improved carcass feed conversion in Nellore bulls finished in a feedlot [45]. Similarly, supplementation with BX in pasture systems has been associated with increased HCW in Nellore bulls [28]. Yet, several studies support our findings, reporting no significant differences in carcass characteristics when cattle were fed just tannins [26,29,31,44].

In the present study, total fecal starch did not differ among treatments. Thus, despite the inclusion of tannins in the diet, no reduction in fecal starch digestibility was observed. Tannins are known to reduce carbohydrate digestibility by forming hydrogen bonds with macromolecules, limiting enzymatic access [46]. However, since fecal samples in our study were collected at the end of the finishing period, it is possible that ruminal microorganisms had already adapted to the presence of tannins, thereby decreasing the negative effects on starch digestion [46]. A study reported that feeding Angus crossbred with CT from Quebracho showed a decrease in fecal starch at 35 DOF as tannin concentrations increased. However, by the end of the experiment (day 95), no differences were observed among treatments [45]. Additionally, a recent study reported that dairy cows fed *Acacia mearnsii* tannins at increasing concentrations (0, 0.14, 0.29, and 0.43% of diet DM) showed higher total-tract apparent digestibility of starch as tannin levels increased [47]. One potential explanation for this improvement is that enhanced starch digestibility may be associated with reduced nitrogen losses through urine [48]. Therefore, these results highlight that the effects of tannins on starch digestibility may vary depending on tannin source, concentration, and feeding period.

Liver abscesses are a significant issue in the United States, with a prevalence ranging from 12% to 32% in commercial feedlot cattle [2]. In addition, the incidence of LA can vary depending on the cattle type. Holstein-fed cattle present a more frequent incidence (25%) compared to fed-beef steers (18.2%) and heifers (19.1%) [49]. Moreover, observations of beef × dairy crossbred populations have shown a greater prevalence of LA, which has been a concern to the cattle industry [50]. New theories about LA formation have been explored in recent years, suggesting that LAs are not only linked to rumen acidosis but also to disruptions in lower gut health. A study demonstrated that microbial communities found in LA are closely related to those in the hindgut, suggesting that LA can be formed by bacterial populations from the lower gut rather than just the rumen [51]. This aligns with the new hypothesis that liver abscesses are not solely the result of rumen acidosis but also involve bacterial translocation from the lower gut due to gut barrier dysfunction. Tylosin phosphate has traditionally been the primary feed additive used to control LA in cattle. However, because of its shared use of antibiotic classification and the associated restrictions, there is a growing pressure for alternative solutions. A study demonstrated that reducing the duration of tylosin phosphate administration did not impact total liver abscesses or performance in feedlot cattle compared to cattle fed for a long period, suggesting a potential reduction in antibiotic usage [52]. Regarding alternatives to antibiotics, a recent study reported that supplementing cattle with a commercial blend of tannins (Silvafeed ByPro) reduced the prevalence of LA compared to sodium monensin [53]. However, other studies showed that tannin supplementation did not affect LA prevalence [29,31,33]. Our study demonstrated that MON + TYL had the lowest prevalence of LA (18.3%), followed by MON + BX (28.5%), BX (36.8%), and CTL (43.1%). The reduction in liver abscesses observed in steers fed MON + TYL is consistent with the literature, where tylosin phosphate is widely recognized for its efficacy in controlling liver abscesses in cattle [8,9]. A classical study reported that liver abscess incidence decreased from 27% to 9% when cattle were fed tylosin alone or in combination with monensin, whereas monensin alone had no effect on liver abscess prevalence [54]. Also, the control of LA is dependent on the use of TYL [7]. Notably, the treatment MON + BX had the second-lowest LA prevalence and could serve as a safe alternative to tylosin in natural and organic marketing programs that restrict or prohibit antibiotic use. Feeding MON + BX may enhance animal efficiency while reducing the incidence of LA, making it a valuable option for programs seeking to minimize the use of tylosin. Also, LA is not only associated with rumen health but also with the overall condition of the gastrointestinal tract (GIT) [2]. Recent microbial community sequencing demonstrated that the *Bifidobacterium* spp. were more abundant in the rumen and ileum of cattle without LA compared to those with LA [51]. These bacteria are related to healthy GIT, emphasizing the importance of maintaining gut integrity and the link with LA. Tannins may also improve the intestinal barrier function in chickens by upregulating the expression of tight junction proteins, mucin proteins, and nutrient transporters in the jejunum [55]. A study found that chickens receiving chestnut tannins during the growing phase had the highest villus-to-crypt ratio in the small intestine [56]. One potential explanation for the decreased LA incidence in animals fed MON + BX is that tannins may enhance the GIT barrier, reducing the translocation of bacteria, such as *Fusobacterium necrophorum* and *Salmonella enterica*, into the bloodstream and reaching the liver. Tannins also may improve nutrient absorption in the small intestine, contributing to overall gut health and immune function. A study reported that tannins extracted from chestnut and quebracho wood have shown strong antibacterial activity against *Salmonella enterica* isolated from poultry [57], indicating their potential as a natural strategy to help control pathogens implicated in liver abscess development. Additionally, higher levels of phenolic compounds in muscle tissue were observed when Holstein steers were fed tannins as an additive, which may suggest that tannins not only influence the GIT but can cross the intestinal barrier and reach other organs [33]. Despite growing interest in natural feed additives, there is still a limited understanding of the mechanisms by which tannins and saponins may influence liver abscess formation, highlighting the need for further research.

It is important to note that differences in cattle type, feeding strategies, harvest weight, and supplement dosage may help explain the variation in responses observed both in our study and in others. While the present study was conducted using steers, many studies from Latin America have used different breeds and intact bulls, which may respond differently to dietary tannins. In addition, differences in diet composition and days on feed could also influence the effects of tannins. The dose of the tannin blend used in our study may have been lower than what is required to see consistent performance benefits in U.S. feedlot cattle. These factors, also cited by Bowman-Schnug et al. [33], should be considered when comparing results across studies using tannin supplementation.

## 5. Conclusions

Overall, these findings in this study indicate that BX alone is not a viable replacement for conventional additives in US feedlot systems, since it did not improve the parameters analyzed. The combination of MON + TYL was the most effective strategy for reducing liver abscess incidence. Similarly, the combination of MON + BX had a moderate reduction in liver abscesses while maintaining feed efficiency comparable to MON + TYL. Therefore, MON + BX may serve as a potential alternative to tylosin phosphate in feedlot programs aiming to reduce antibiotic use. However, further research is needed to validate these effects under different production conditions, including variations in cattle sex, breed, dosage, and environmental factors. Additionally, exploring the mechanisms underlying the potential synergism between MON and BX will be critical to understanding its broader application.

## Figures and Tables

**Table 1:** Feed ingredients and chemical composition of the basal diet during the finishing phase.

Item	Finishing Diet
Ingredients, % of DM ^1^	
Flaked corn	59.62
High-moisture corn	21.14
Triticale silage	4.26
Supplement ^2^	3.82
Palm oil	3.48
Distillers grains	2.95
Wheat straw	2.46
Alfalfa hay	2.27
Nutrient content, % of DM ^3^	
Dry matter	73.22
Crude protein	11.80
Non-protein nitrogen	2.42
Acid detergent fiber (ADF)	6.86
Neutral detergent fiber (NDF)	13.48
peNDF ^4^	6.57
Crude fat	6.91
NE_m_^5^, Mcal/kg	2.47
NE_g_^6^, Mcal/kg	1.56
Ca	0.71
P	0.31

^1^ Dry matter; ^2^ Supplement was formulated to include 14.29% calcium, 2.00% potassium, and 1.07% magnesium on DM basis; ^3^ Ration analyses were conducted using wet chemistry by a commercial laboratory (Servitech, Dodge City, KS, USA); ^4^ Physically effective NDF; ^5^ Net energy for maintenance; ^6^ Net energy for gain, Mcal.

**Table 2:** Feedlot performance of steers supplemented with a proprietary tannin blend, with or without sodium monensin, or with the combination of sodium monensin and tylosin phosphate during the finishing period.

	Treatment ^1^					
	CTL	BX	MON + TYL	MON + BX	SEM ^2^	*p*-Value
Shrunk body weight						
Initial, kg	254	254	254	254	9.2	0.499
Reimplant, kg	496	494	496	499	10.3	0.265
Carcass-adjusted final ^3^, kg	637 ^ab^	632 ^b^	639 ^ab^	642 ^a^	4.7	0.002
ADG						
Overall, kg	1.67 ^ab^	1.65 ^b^	1.68 ^ab^	1.69 ^a^	0.053	0.002
D0 to reimplant, kg	1.82	1.81	1.83	1.85	0.096	0.294
Reimplant to harvest, kg	1.44	1.41	1.47	1.46	0.033	0.305
DMI						
Overall, kg	9.34 ^a^	9.18 ^ab^	8.94 ^c^	9.03 ^bc^	0.179	<0.001
D0 to reimplant, kg	8.98 ^a^	8.84 ^ab^	8.50 ^c^	8.71 ^b^	0.256	<0.001
Reimplant to harvest, kg	9.79 ^a^	9.61 ^ab^	9.50 ^ab^	9.43 ^b^	0.189	0.005
F:G						
Overall	5.60 ^b^	5.58 ^b^	5.34 ^a^	5.35 ^a^	0.096	<0.001
D0 to reimplant	4.94 ^b^	4.90 ^b^	4.66 ^a^	4.71 ^a^	0.124	<0.001
Reimplant to harvest *	6.85	6.82	6.48	6.48	0.173	0.105

^1^ CTL, control; BX, blend of tannins and saponins; MON + TYL, monensin + tylosin phosphate; MON + BX, monensin + blend of tannins and saponins. ^2^ Standard error of mean. ^3^ Calculated assuming a 64% dress. * Non-parametric Kruskal–Wallis model used for analysis because of violated assumptions. ^a,b,c^ Treatment effect, within a row means without a common superscript letter differ (*p* ≤ 0.05).

**Table 3:** Health outcomes of steers supplemented with a proprietary tannin blend, with or without sodium monensin, or with the combination of sodium monensin and tylosin phosphate during the finishing period.

	Treatment ^1^					
	CTL	BX	MON + TYL	MON + BX	SEM ^2^	*p*-Value
Pens, *n*	12	12	12	12		
Head per treatment	745	747	750	744		
Morbidity, %	5.31	5.76	5.77	5.00	1.046	0.898
Respiratory morbidity, %	4.02	3.87	4.41	3.50	0.958	0.847
First treatment, %	3.54	3.08	3.77	3.02	0.874	0.836
Second treatment, %	0.30	0.13	0.40	0.25	0.279	0.810
Third treatment, %	0.00	0.15	0.29	0.13	0.205	0.911
Bloat, %	0.48	0.73	0.29	0.27	0.205	0.559
Mortality, %	0.97	1.75	1.02	0.94	0.501	0.452
Respiratory, %	0.32	1.02	0.73	0.67	0.384	0.537
Digestive, %	0.48	0.73	0.29	0.13	0.325	0.390

^1^ CTL, control; BX, blend of tannins and saponins; MON + TYL, monensin + tylosin phosphate; MON + BX, monensin + blend of tannins and saponins. ^2^ Standard error of mean.

**Table 4:** Carcass characteristics of steers supplemented with a proprietary tannin blend, with or without sodium monensin, or with the combination of sodium monensin and tylosin phosphate during the finishing period.

	Treatment ^1^					
	CTL	BX	MON + TYL	MON + BX	SEM ^2^	*p*-Value
HCW, kg	408 ^ab^	404 ^b^	409 ^ab^	411 ^a^	3.0	0.001
Yield grade	2.80 ^ab^	2.73 ^b^	2.94 ^a^	2.93 ^a^	0.215	0.004
Ribeye area, sq cm	93.6	93.5	93.2	92.5	1.65	0.198
Fat thickness, cm	1.55 ^ab^	1.51 ^b^	1.57 ^ab^	1.59 ^a^	0.134	0.035
KPH, %	3.34	3.21	3.38	3.30	0.211	0.060
Marbling score ^3^	484 ^ab^	479 ^b^	487 ^ab^	497 ^a^	11.7	0.046
Quality grade distribution *						0.073
Prime, %	2.9	1.7	2.6	2.8	0.89	
Choice, %	79.3	77.3	81.2	81.7	2.85	
Select, %	15.3	16.2	12.5	12.0	3.54	
Liver score distribution *						<0.001
Abscess prevalence, %	43.1 ^a^	36.8 ^a^	18.3 ^c^	28.5 ^b^	2.49	<0.001
A+ prevalence, %	28.7 ^a^	25.7 ^a^	12.0 ^c^	17.9 ^b^	1.92	<0.001
A prevalence, %	14.2 ^a^	11.0 ^a^	6.2 ^b^	10.5 ^a^	1.65	<0.001

^1^ CTL, control; BX, blend of tannins and saponins; MON + TYL, monensin + tylosin phosphate; MON + BX, monensin + blend of tannins and saponins. ^2^ Standard error of mean. ^3^ Small^00^ = 400, Modest^00^ = 500. ^a,b,c^ For the treatment effect, within a row means without a common superscript letter differ (*p* ≤ 0.05). * Distribution tested by χ^2^ test statistic.

## Data Availability

The data that support the findings of this study are available from the authors on request.
